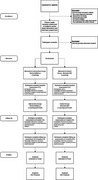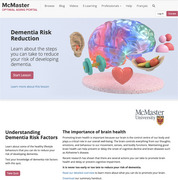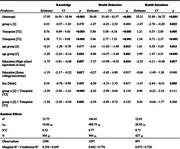# Evaluating the Impact of a Web‐Based Educational Intervention on Dementia Risk Factor Awareness, Intentions, and Health Behaviours: Results from a Mixed‐Methods Randomized Controlled Trial

**DOI:** 10.1002/alz70863_110552

**Published:** 2025-12-23

**Authors:** Anthony J. Levinson, Stephanie Ayers, Sandra Clark, Rebekah Woodburn, Maureen Dobbins, Dante Duarte, Roland Grad, Dima Hadid, Nick Kates, Doug Oliver, Alexandra Papaioannou, Sharon Marr, Karen Saperson, Amy Schneeberg, Henry Siu, Gillian Strudwick, Richard Sztramko, Sarah Neil‐Sztramko

**Affiliations:** ^1^ McMaster University, Hamilton, ON Canada; ^2^ McGill University, Montreal, QC Canada; ^3^ Independent Statistician, Vancouver, BC Canada; ^4^ CAMH, Toronto, ON Canada

## Abstract

**Background:**

Dementia is a major global health challenge, with prevention strategies increasingly focusing on modifiable risk factors. Despite evidence that lifestyle changes can reduce dementia risk by up to 45%, public awareness remains low. Web‐based platforms offer scalable solutions to bridge this knowledge gap. This study evaluated whether exposure to DementiaRisk.ca improves knowledge of dementia risk factors, intention to engage in risk‐reduction behaviors, and actual health behaviors.

**Method:**

A sequential explanatory mixed‐methods design was employed. 510 participants were randomized into intervention (*n* = 265) or control (*n* = 245) groups. The intervention group received DementiaRisk.ca, which included a 35‐minute multimedia lesson on dementia risk reduction and brain health and micro‐learning emails. Control participants received a comparable lesson and micro‐learning emails on mild cognitive impairment. Outcomes (knowledge, health behavior intentions, and behavior change) were measured at baseline (T1), 4 weeks (T2), and 12 weeks (T3). Qualitative feedback surveys were collected to explore experiences and barriers/facilitators.

**Result:**

Participants were predominantly older adults ≥55 (55%), female (61%), and reported good to excellent health (81%). Both groups showed increases in knowledge, with the intervention group demonstrating significantly larger gains (mean increase of 8.8 points vs. 6.2 in control, *p* <0.01). These differences persisted at T3 (mean difference ‐1.54, 95% CI [‐2.50, ‐0.58]). Participants with lower education levels showed the greatest knowledge gains. Both groups had significant increases in intentions to adopt healthy behaviors, sustained through T3. The intervention group showed more substantial improvements in health behaviors, with a 5.88‐point increase at T2 and a sustained 5.06‐point increase at T3. Qualitative feedback indicated strong engagement, with participants reporting lifestyle changes (i.e., increased physical activity, dietary improvements, increased doctor visits). Barriers included technological issues and time constraints. Younger participants were more prevention‐focused, while older participants sought information on dementia management and caregiving.

**Conclusion:**

DementiaRisk.ca significantly improved knowledge of dementia risk factors and promoted behavior changes, especially among individuals with lower education levels. These findings suggest the platform's potential to reduce health inequities and improve public health outcomes in dementia prevention. Future research will focus on optimizing engagement and expanding reach to diverse populations, with an emphasis on long‐term impact.